# Early and late effects of absorbable poly(vinyl alcohol) hernia mesh to tissue reconstruction

**DOI:** 10.1049/nbt2.12015

**Published:** 2021-02-02

**Authors:** Daniella Fehér, Andrea Ferencz, Györgyi Szabó, Krisztina Juhos, Domokos Csukás, Constantinos Voniatis, Lilla Reininger, Kristóf Molnár, Angéla Jedlovszky‐Hajdú, György Wéber

**Affiliations:** ^1^ Department of Surgical Research and Techniques Semmelweis University Budapest Hungary; ^2^ Department of Pathology and Experimental Cancer Research Semmelweis University Budapest Hungary; ^3^ Laboratory of Nanochemistry Department of Biophysics and Radiation Biology Semmelweis University Budapest Hungary

## Abstract

Hernia is a defect of the abdominal wall. Treatment is principally surgical mesh implantation. Non‐degradable surgical meshes produce numerous complications and side‐effects such as inflammatory response, mesh migration and chronic pain. In contrast, the biodegradable, poly (vinyl alcohol) (PVA) based polymers have excellent chemical, mechanical and biological properties and after their degradation no chronic pain can be expected. The toxicology of PVA solution and fibers was investigated with Human dermal fibroblast‐ Adult cell line. Implantation tests were observed on long‐term contact (rat) and large animal (swine) models. To measure the adhesion formation, Diamond and Vandendael score were used. Macroscopical and histological responses were graded from the samples. In vitro examination showed that PVA solution and fibers are biocompatible for the cells. According to the implantation tests, all samples were integrated into the surrounding tissue, and there was no foreign body reaction. The average number of adhesions was found on the non‐absorbable suture line. The biocompatibility of the PVA nanofiber mesh was demonstrated. It has a non‐adhesive, non‐toxic and good quality structure which has the potential to be an alternative solution for the part of the hernia mesh.

## 1 INTRODUCTION

The issue of abdominal wall defect and its repair has been a main research topic both in the field of surgery and nanomedicine over the past years. In general, an incisional hernia can occur in more than 20% of patients after abdominal surgery [1, 2, 3]. It can appear anywhere in the body where muscle and fascia layers are weaker and thinner. The abdominal hernia may be congenital or acquired, whereby an abdominal organ (or its contents) protrudes through this hole. There are many factors which can play a role in the development of the disease, such as being overweight, prolonged heavy physical activity, general condition of the patient, materials used during surgery or even persistent increase in abdominal pressure (persistent coughing, constipation, pregnancy, etc.). To treat the defect of the abdominal wall, several strategies have been developed.

Suture repair techniques have dominated ventral hernia repair over a century. The most popular of these techniques was the Mayo duplication. In larger hernias, suture repair requires the application of tension to the fascia in order to close the orifice. Therefore, many suture repairs failed mechanically, and recurrence rates were found to be as high as 54%. The advantages of mesh implantation were first confirmed in an influential trial by Luijendijk et al., who found recurrence rates to be nearly halved by using mesh compared to suture repair [4, 5, 6].

During surgery, the muscle reinforcement techniques always apply various synthetic, non‐absorbable materials under reconstruction. The routinely used techniques where the mesh is placed either over or under the defect often called ‘tension free’ repair methods [7, 8, 9].

An optimal mesh has to be biocompatible, with excellent biomechanical properties, and it should not cause any inflammatory response. In recent years non‐degradable polypropylene (PP) is the most widely used hernia mesh in large incisional defect, which is often cause serious complications, such as infection, adhesion formations [10]. Because of these complications, researchers have to combine or replace them with nanomaterials.

Biocompatibility studies of each degradable material require complex experiments both in vitro and under different in vivo conditions. Because of the sensitivity and reproducibility of the cells, in vitro cell culture tests are often used to screen the systemic effects of implants. The most important thing is the biological response of the host living system. This could appear during incorporation of any foreign implants.

Besides, these in vivo hernioplasty studies could model in different ways when a tissue is injured by implanted materials or the changes of the implant properties during its degradation [11, 12]. Adhesion formation to the mesh could be easily studied using our experimental models.

Most of these materials have been known in the medical field before their appearance in nanomedicine, such as poly(vinyl alcohol) (PVA) which is well‐known for being biocompatible, biodegradable and have stable molecule structure with non‐toxic fragments. PVA was discovered in the 1920s and was used for fibre coating, films for packing or in adhesives polymerisation. For the formation of the scaffold's porous structure, several different methods have been described in the literature, for example, nanofibre self‐assembly, textile technology, gas foaming, freeze drying, electrospinning, etc. [13, 14]. In our electrospinning process, a high electric field is generated between a polymer solution held by its surface tension at the end of a syringe (or a capillary tube) and a collection target [15, 16, 17, 18].

The morphology of the mesh fibres is affected by various parameters, including viscosity, molecular weight, surface tension, such as humidity and temperature of the environment. Recent studies have revealed that biomaterials need special physical, chemical and mechanical properties [19, 20]. Moreover, introducing new degradable devices for hernia or skin replacement requires novel developments [21].

In our research project the objectives were to develop and optimized a novel PVA hernia mesh, which was dissolved in water and the concentration of PVA aqueous solution was varied from 5 to 15 weight %. The cell cultures, using Human dermal fibroblasts (HDFa) are conducted to assess the viability and potential application of PVA hydrogel as a mesh for hernia repair. In our large animal model absorbable PVA polymer membranes were implanted laparoscopically, on the right side on the abdominal wall without creating any abdominal wall defect. As self‐control in each animal polypropylene meshes were placed on the left side with the same protocol. Macroscopic findings showed a new mesothelial layer (as a new peritoneum) on PVA meshes and they were integrated to the host tissue. In contrast, PP meshes inducted strong adhesion in the same abdomen. The present study evaluated the capability of abdominal regeneration and anti‐adhesion properties on our PVA fabric after its intra‐abdominal placement on the peritoneum. This synthetic polymer could be a promising scaffold for biomedical application, easily accessible, has good mechanical properties which can lead to complications such as adhesion formation.

## 2 MATERIALS AND METHODS

### 2.1 PVA mesh synthesis and sterilization

**FIGURE 1:**
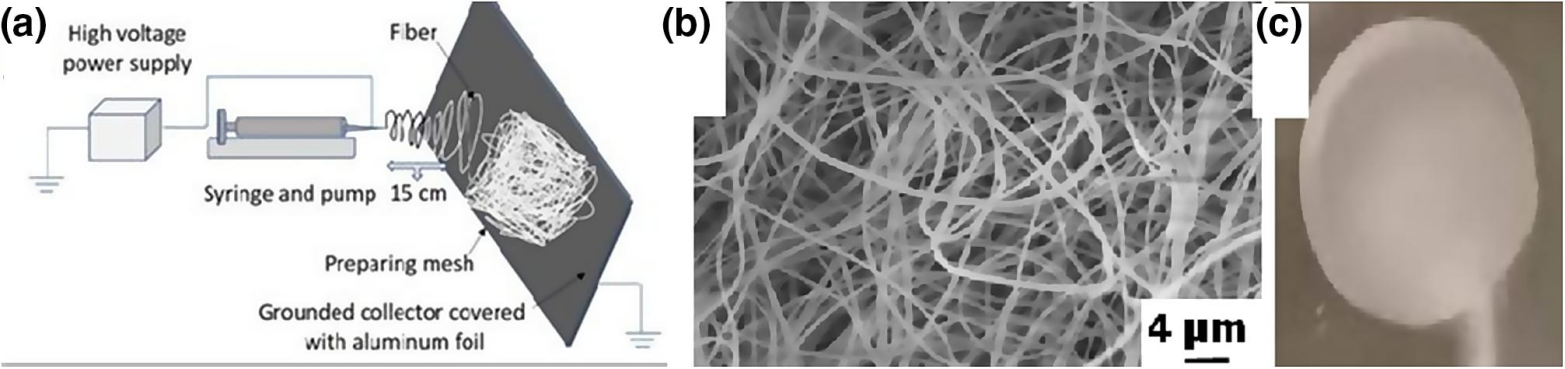
Schematic figure of electrospinning (a), SEM photo of the PVA fibres (b), suturable PVA nanomesh (c)

PVA biomimetic scaffold (Figure 1c) with suturable profile and good mechanical properties was manufactured by our research group. PVA powder (*M*_w_ ∼ 72,000) was purchased (Merck‐Scuchardt, Hohenbrunn, Germany) and for its formation, conventional electrospinning technique was used as our research team previously described [22]. Briefly, PVA was dissolved in water at 90°C for 2 h and maintained for 30 min to ensure homogenization. Concentration of PVA aqueous solution was varied from 5 to 15 weight %. PVA solution was placed into a glass syringe (Fortuna Optima, Sigma‐Aldrich, USA), and an electrode from high voltage power supply (16–20 kV) was attached to a metal Hamilton syringe tip. This technique relies on a DC applied potential between a syringe tip and a substrate, typically an aluminium foil, which was located on the surface of a grounded collector. The synthetized mesh showed a random, crosslinked, and compact structure with 1 mm thickness (Figure 1a). In our current setup the residual solvent was water, which does not cause any side effect.

Before it is used in cell culture or in an animal model the meshes were sterilized in chlorine dioxide (ClO_2_) solution (Solumium, Sanitaria, Budapest, Hungary) for 2 h according to the manufacturer's protocol [23]. Only sterilized meshes were used in vitro and in vivo experiments.

### 2.2 Degradation behaviour of the PVA mesh

The surface properties of the PVA mesh were examined using scanning electron microscope (SEM) under 15‐kV accelerated electron beam after being vacuum coating with a thin layer of platinum (Figure 1b).

In vitro degradation tests of the fibrous layer were carried out in phosphate‐buffered saline (PBS) solution containing 2% sodium azide (NaN_3_) under physiological condition (37°C, pH = 7,4). Series of six samples were used to determine their initial weights. The degradation solution was replenished every 15 days. The specimens were removed from the solution at predetermined times (30 and 90 days) and washed with the distilled water before drying at 37°C for 24 h. Each sample was than weighed, and the weight loss was calculated considering the initial dry weight.

### 2.3 Proliferation and toxicology test with HDFa cells

Human dermal fibroblast – Adult (HDFa) cells (Thermofisher Scientific, CA, USA) were cultured until passage five and were seeded onto the top of the PVA nanofibres samples at a concentration of 3 × 10^4^ cells/well. Cells were incubated at 37°C, in a 5% CO_2_ atmosphere incubator, using Medium 106 (Thermofisher Scientific, CA, USA), as specific cell medium. The medium comprised low serum growth supplement (LSGS) without antibiotics. For investigation, cells were cultured on 24‐well plates. The medium was changed every two days to ensure the adequate supply of nutrients in the plates. After 24, 72, 168 h, a subset of scaffolds (*n* = 5) was stained with Vybrant Dio Cell‐labelling Solution (Thermofisher Scientific, CA, USA) for the visualization. Meshes were fixed in formaldehyde solution overnight at 4°C and were dehydrated with ethanol (concentration 60%). The morphology of the attached cells on the surface of the scaffold was analysed. The control group contained HDFa cells and medium without nanofibre samples. Photos were taken with digital camera (DEM 130, Scope Photo software) and fluorescence microscope (Nikon Eclipse 80i).

### 2.4 Cell adhesive test

Cells were seeding and cultured under the same conditions as in ‘Proliferation and toxicology test’ section. Cells in culture medium were counted (Nm) after 72 h of incubation.

The cell adhesion ratio for each condition was calculated using the following equation: Adhesion ratio % = (1 − Nm/3,0 × 10^4^) × 100. All data reported were the mean of three examinations.

### 2.5 Implantation tests

#### 2.5.1 Animals

Biocompatibility and biodegradability of electrospun PVA samples were investigated on long‐term rodent model. Wistar rats (*n* = 48) with average 250 ± 50 g body weight were used for this study. Domestic pigs (*n* = 4) with average 30 ± 5 kg body weight were used as a large animal model. The animals were kept under standard laboratory conditions and had free access to food and water. All animal experimental procedures were conducted according the Hungarian National Food Chain Safety Office (22.1/1244/3/2011.) and followed the protocols approved by our Research Team. Wistar rats were anesthetized by injecting Ketamine (70 mg/bodyweight kg, Calypsol 50 mg/ml inj.) and Xylazine (10 mg/bodyweight kg, CP‐Xylazine 2 % inj. AUV) (Richter Gedeon Ltd, Budapest, Hungary) mixture in 4:1 ratio intraperitoneally. Before surgical intervention domestic pigs were administered premedication with a mixture of Ketamine (5 ml/35 kg bodyweight, Calypsol 50 mg/ml inj.), Xylazine (5 ml/35 kg bodyweight, CP‐Xylazine 2% inj. AUV) and Atropine (1 ml/35 kg bodyweight). For general anaesthesia, intratracheal cannula was used with Isoflurane (2.5%–3.5% v/v) gas in pure oxygen. After mesh implantation, the animals were kept under control. After a given period, they were re‐anesthetized as written above and the tissue reaction such as adhesion formation, tissue integration, dislocalization, seromas, inflammation and mesh shrinkage were evaluated by macroscopic examination. Tissue samples with meshes were taken for histological examination. Samples were placed in 10% buffered formaldehyde solution for long‐term storage. Inflammation cells, fibroblasts and tissue reaction were determined.

### 2.6 Groups

#### 2.6.1 Long‐term contact model

**FIGURE 2:**
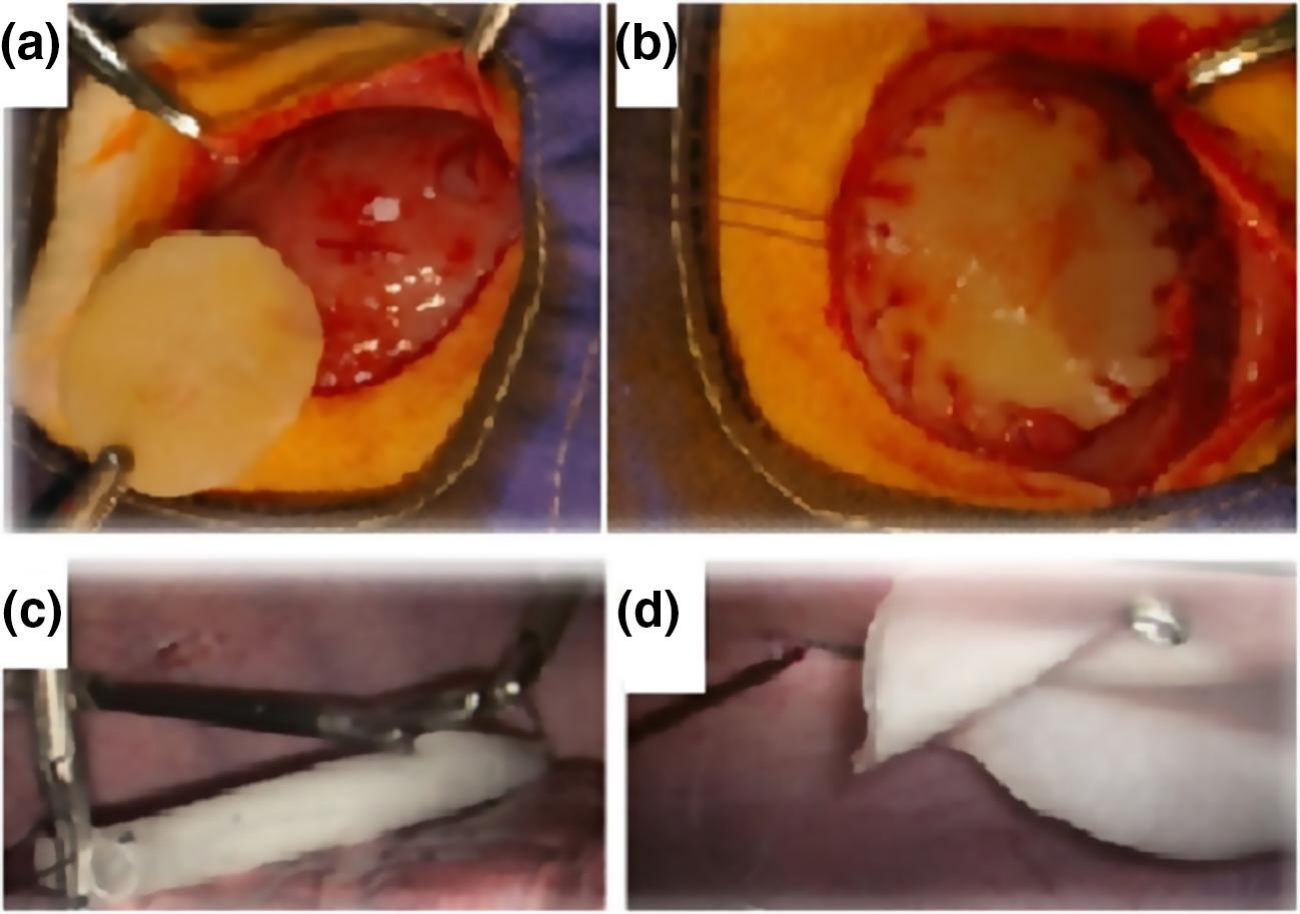
Wall defect in the abdomen, long term animal model (a), placement technique of the novel PVA mesh (b), PVA mesh in large animal model, entered and unfolded PVA mesh in the abdominal cavity (c), final results: implanted and fixed PVA mesh (d)

From a total of 48 adult male Wistar rats (250‐300 g), forty animals underwent mesh implantation. They were divided into four groups: group I (GI), group II (GII), group III (GIII) and group IV (GIV) randomly. After left‐side transrectal laparotomy, the tissue defect on the right side was covered, in an onlay method, with 2.5 cm piece of PVA mesh (*n* = 30) and fixed with a 4/0 polypropylene (PP) suture (Figure 2a,b). The skin was closed with simple interrupted suture line used 3/0 PP suture material.

Reoperation was performed on the given postoperative days (POD): on 7th POD (*n* = 10), 14th POD (*n* = 10), 28th POD (*n* = 10) and 90th POD (*n* = 10). Each mesh was rated independently by two investigators. Adhesions were assessed semi‐quantitatively using scoring system established by Diamond (grade 0 meant 0% adhesions, while grade 4 showed more than 75% adhesions) (Table 1). To make comparison easier with other articles, adhesion formation was scored by Vandendael score as well, where the amount of individual adhesion strands, their width, thickness and strength was counted. Each parameter should be rated with 1–3 score points. A total of amount 1–4 points are regarded as mild grade of adhesion, 5–8 points as moderate, 9–12 points as severe (Table 1). According to the adhesion formation, three types were established. Columnar (the adhered surfaces less than 0.5 × 0.5 cm), curtain‐like (the adhered surfaces 0.5 cm <and 0.5 cm>) and large surface cohesive (the adhered surfaces more than 0.5 × 0.5 cm) (own score system).

**TABLE 1:** Vandendael and Diamond scale score

Score	Extent (%)	Tenacity	Type	Width of Adhesion (mm)	Thickness (mm)	Subjective Strength (+)
0	0	None	None	‐	‐	‐
1	<25	Easily lysed	Filmy, no vessels	<2	<1	+
2	25–50	Lysed with traction	Opaque, no vessels	2–10	1–3	++
3	50–75	Required sharp dissection	Opaque, small vessels	>10	>3	+++
4	>75	Required sharp dissection	Opaque, large vessels	‐	‐	‐

Vandendael: Grade 1 = mild (1–4), Grade 2 = moderate (5–8), Grade 3 = severe (9–12)

The main purpose was to investigate the biocompatibility by measuring early and long‐term signs of adhesion formation and inflammatory response. In the control animals (*n* = 8), only a 4 cm long median laparotomy was performed than it was closed with simple interrupted suture line (3/0 PP).

### 2.7 Large animal model – 5 weeks' follow‐up

Pilot study was performed on porcine model (*n* = 4) without creating abdominal wall defect. Absorbable PVA (*D* = 8 cm) and non‐absorbable polypropylene (PP) meshes (*D* = 8 cm) were implanted laparoscopically (Figure 2c and fixed intraperitoneally (Figure 2d) into each animal on left and right sides symmetrically with respect to the linea alba. PVA meshes were not sticky and their handling was not different from PP meshes. The meshes were secured to the muscle inferiorly to the four corners with 5 mm fasteners by Protack Fixation Device (Covidien, Dublin, Ireland). Five weeks after the surgery, all swine were euthanized, and tissue‐mesh samples were removed. Explanted specimens contained mesh, abdominal muscle, fascia and skin. For each specimen, pieces measuring 3 × 3 cm were used for histology analysis and pieces 1 × 1 cm were cut for microscopical evaluation.

### 2.8 Tissue integration and mesh dislocation

Ingrowth of the mesh into the surrounding area was studied by lifting the mesh with forceps after the reoperation. Excellent integrated implant (tissue ingrowth of >75% of mesh) was scored as 1, well‐integrated implant (up to 75% of the surface) was scored as 2, whereas moderate integration (no tissue ingrowth, less than 50% of the surface) was scored as 3. Dislocalization of the meshes was studied after the reoperation. If the mesh was in original place, the score was 1, if the mesh dislocated the score was 2.

### 2.9 Histological evaluation

The implanted mesh samples with surrounding tissues were preserved and sent for histological evaluation. Samples containing the mesh and all layers were embedded in paraffin. Four‐micrometre sections were cut and stained with Hematoxylin and Eosin (H&E). Glass slides were scanned with Panoramic Scan (3DHISTECH, Budapest, Hungary) using Plan‐Apochromat 20× magnification objective, a 1.6× camera adapter magnification and 1× Optovar magnification with a CIS VCC‐FC60FR19CL camera, resulting in 0.24 µm/pixel resolution. The inflammatory response was quantified according to the type and intensity of the reaction. The evaluation was done in a ‘blind’ manner by involving two independent pathologists.

#### 2.9.1 Statistical analysis

All quantitative results were expressed as means ± standard deviation (*n* = 5). Data were analysed with statistically significant values defined as *p* < 0.05 based on Student's *t*‐test and one‐way ANOVA were used. A *p* value of less than 0.05 was assumed to indicate statistically significant difference.

## 3 RESULTS

### 3.1 Results of the degradation behaviour

According to SEM, electrospinning yielded smooth and fairly uniform fibres with an average diameter of 390 ± 20 nm (Figure 1b). Each sample lost 1 mm from their length. And the loss of the weight was calculated considering the initial dry weight.

### 3.2 Results of the proliferation and toxicology test with HDFa cells

Cells in different conditions (with and without PVA scaffolds in culture medium) were counted (Nm) after 24, 72 and 168 h of incubation. Cell adhesive test showed that adhesion ratio was not significantly higher after 168 h without PVA scaffolds than with the presence of PVA.

**FIGURE 3:**
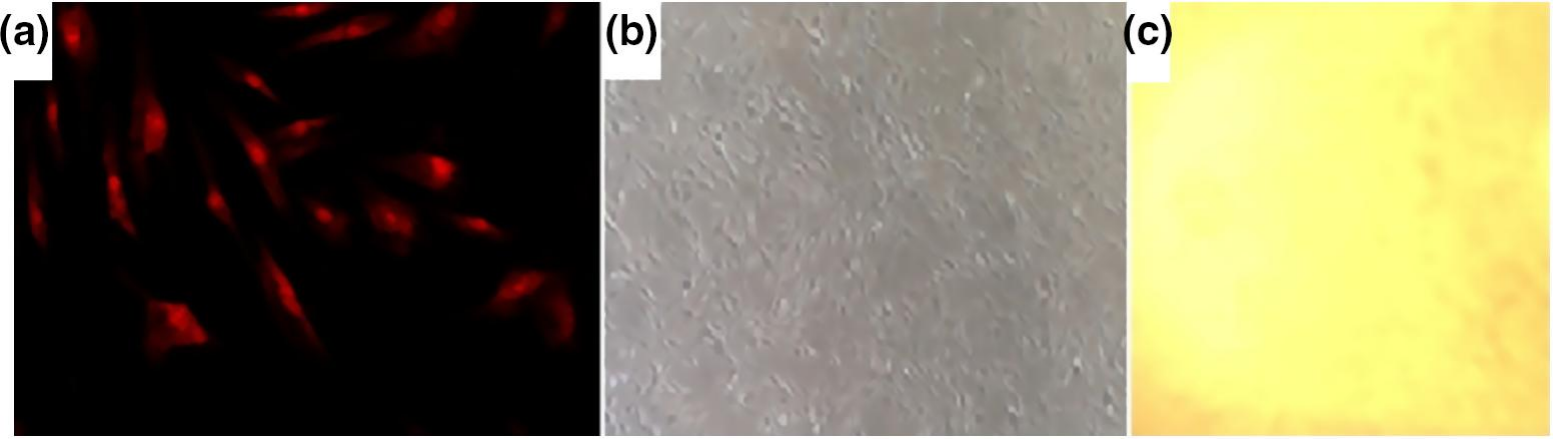
HDFa cells with PVA membranes: confocal micrograph of HDFa cells in Medium 106 with PVA membrane after 72 h incubation (a), HDFa cells with PVA mesh device after 168 h (b), cell‐ingrowth onto the edge of the PVA mesh (c)

We found that HDFa (Figure 3a,b) cells had normal, healthy shape and cells could proliferate with the PVA hydrogels and on the PVA meshes. After the 24 h period, cells had normal and healthy shape, only a few spherical cells were observed in the medium. And after the 168 h incubation time, the cells were confluent and showed an excellent biocompatibility. The examination of PVA meshes resulted that the cell infiltration was relatively poor on the surface of the meshes (Table 2) which is a good sign for their non‐adhesive quality. The cellular responses indicate no signs of toxicity. After 168 h we found that the cells could easily attached to the bottom of the wells and just a few cells grow onto the edge of the scaffolds suggesting the cell migration (Figure 3c and Table 2).

**TABLE 2:** Proliferation and toxicology test on PVA meshes, conducted 24‐well plates with HDFa cell line

Samples	Attachment/Morphology	Attachment/Morphology 2	Attachment/Morphology 3
Attached to the surface	+/−	−/−	−/−
Cells under the mesh	+/+	+/−	+/+
Control	+/+	+/+	+/+

(+) cells attached and proliferated on the surface; (−) cells did not attach, circular floating cells; (+/−) mixed results

According to our results, The PVA meshes did not have any toxic effects for different cell lines and we observed that PVA meshes prevent the adhesion of the cells to the surface. Therefore, we conclude that this mesh can be a great potential for tissue engineering especially for hernia repair.

### 3.3 Results of the implantation tests

All surgical interventions were performed without any difficulties. There were no post‐operative mortalities apart from one animal which was terminated because of technical issues. Wound healing was normal without any complications. In the long‐term (rat) and large animal (swine) models, the purpose was to monitor the host tissue response. After dissection of the animals, scaffolds were found with no signs of inflammatory response or strong foreign body reaction.

### 3.4 Results of long‐term contact model

**FIGURE 4:**
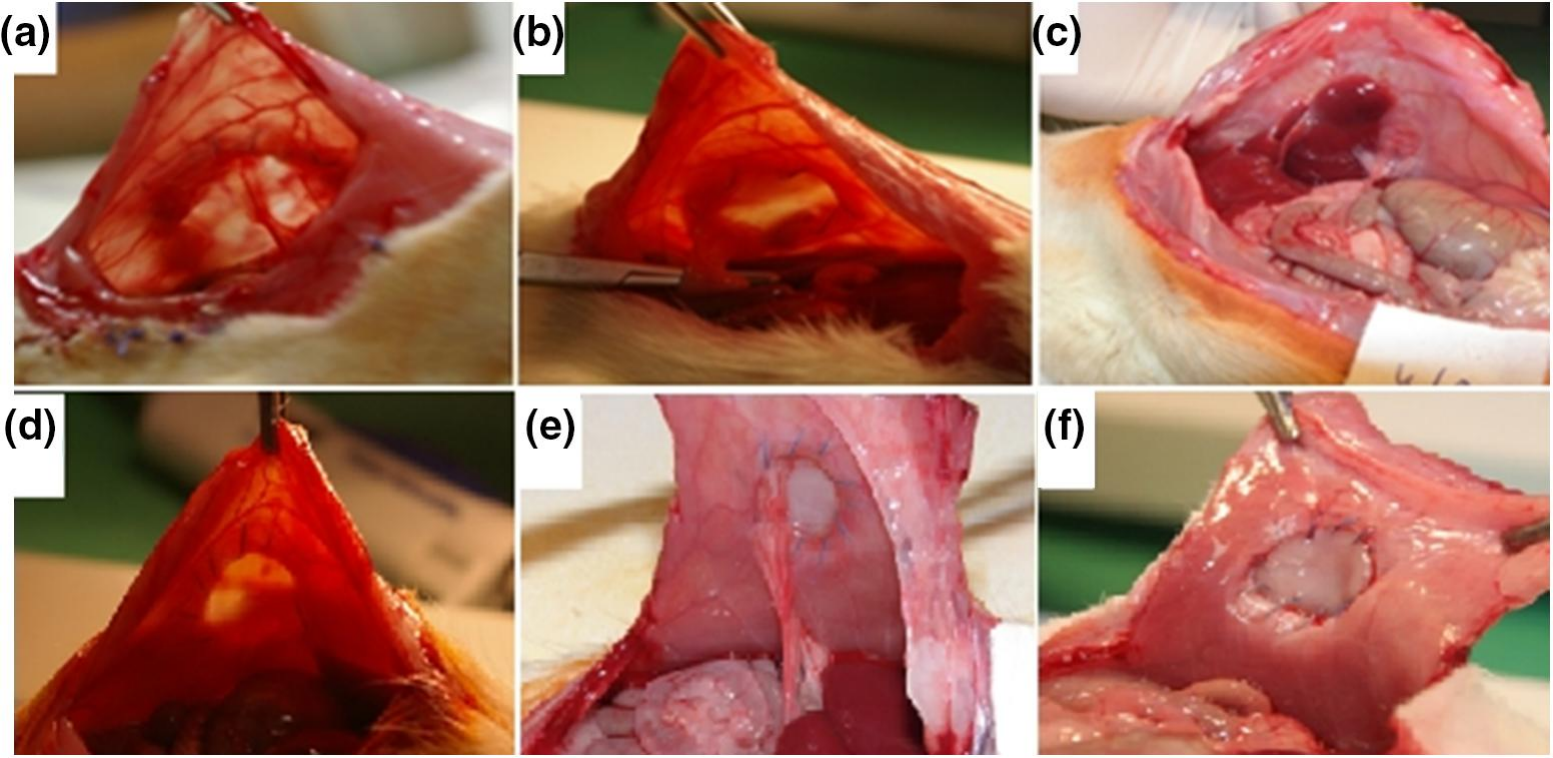
Early postoperative results: columnar adhesion (a), curtain‐like adhesion (b), and large surface adhesion (c) in rat models. Columnar adhesions around the suture line are visible at an early stage, on the 14th POD (d). On the 28th POD fibrotic scar behind the PVA mesh completely integrated (e). There was not found any post‐operative complications on the 90th POD (f)

In rat model all three forms of adhesion appeared around the implanted absorbable mesh. In the early postoperative period, on the 7th POD in GI, both curtain‐like adhesions (Figure 4a) and columnar adhesions (Figure 4b), in GII, on the 14th POD large surface adhesions (Figure 4c) were detected during tissue biopsies. Two weeks later peri‐implant adhesions were not detectable but surrounding the suture line significant columnar adhesions were produced by non‐absorbable suture materials. In the later postoperative phase, GIII and GIV, mesh completely integrated into surrounding tissues, absorbed, and left a fibrotic scar (Figure 4f).

After the exploration of the abdominal wall the PVA mesh samples were evaluated macroscopically. Infection was not found in the environment of PVA meshes in none of the animals. In two animals some serous fluid could be detected between the PVA mesh and the skin. During the determination of the adhesion localization, it was found that most of the adhesions were attached to the non‐absorbable suture material (*n* = 19) which was used for fixing the mesh to the abdominal wall and not to the surface of the PVA mesh (*n* = 5). The adhesion attached to the mesh surface was less than 30% of the total surface in all cases. Great omentum was the main tissue that took part in the adhesion formation in each animal. The adhesion involves liver in some cases (*n* = 6) because of the proximity of it. Stronger traction needed for removing the moderate stable adhesion (*n* = 13), in six cases the adhesion was instable, especially in the early postoperative days. Early postoperative results were found columnar adhesion (Figure 4a), curtain‐like adhesion (Figure 4b), and large surface adhesion (Figure 4c) in rat models. Columnar adhesions were seen around the suture line, there were visible at an early stage, on the 14th POD (Figure 4d) and fibrotic scar behind the PVA mesh on the 28th POD completely integrated (Figure 4e).

In eight animals' stable adhesions could be found where the attached surfaces could not be pulled apart from each other at all, no matter how strong the pull was. Adhesion formation was barely present in the control group. There were only two animals where columnar adhesion could be found. In both cases, omentum attached to the incision line with a very fine surface. In GI, the results were similar. Adhesion formation was minimal except for three cases where organs induced larger adhesion. PVA meshes were integrated into the surrounding area. In the most cases (GII, GIII), adhesion was only found on the Polypropylene suture line**.** It was evaluated and classified on the Diamond Scale and Vandendael score (Table 3). The scaffolds did not lose their size, weight, or composition by the end of the experiments.

**TABLE 3:** Results of adhesion formation and its histological evaluation by Diamond and Vandendael score in small animal model

Score	Number of Animals (*n*)	Extent (%)	Tenacity	Type
Diamond				
0	7	0	None	None
1	25	<25	Easily lysed	Filmy (no vessels)
2	13	25–50	Lysed with traction	Opaque (no vessels)
3	2	51–75	Required sharp dissection	Opaque (small vessels)
4	1	>75	Required sharp dissection	Opaque (large vessels)
**Vandendael**	**Animals (*n*)**	**Width of Adhesion (mm)**	**Thickness (mm)**	**Subjective Strength (+)**
1	32	<2	<1	+
2	15	2–10	1–3	++
3	1	>10	>3	+++

### 3.5 Results of the large animal model

**FIGURE 5:**
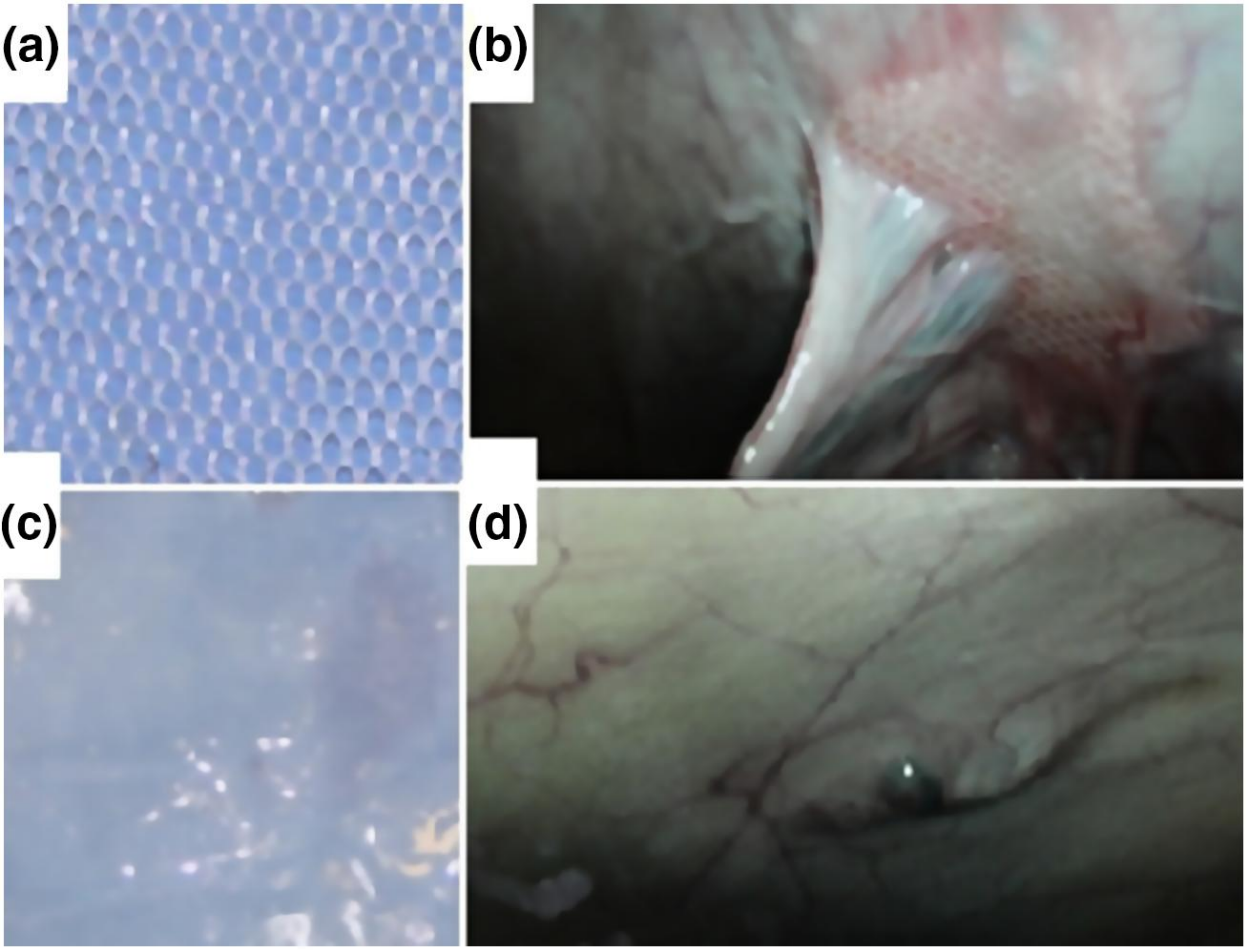
Procurable PP mesh before surgery (a). Laparoscopic view of PP meshes after 5 weeks implantation on the left side of the abdominal wall (b). PVA mesh before implantation (c). PVA mesh on the right side of the abdominal wall after 5 weeks implantation (d)

In large animal model absorbable PVA polymer meshes were implanted laparoscopically, on the right side on the abdominal wall without creating any abdominal wall defect. As a self‐control in each animal polypropylene meshes were placed on the left side with the same protocol (Figure 5). Macroscopic findings showed a new mesothelial layer (as a new peritoneum) on PVA scaffolds and they were integrated to the host tissue. In contrast, PP meshes inducted strong adhesion in the same abdomen. Most of the adhesions connected to PP mesh (Figure 5b), and less frequently with PVA meshes. The mean adhesion for the PVA mesh significantly less than PP meshes. Fewer giant cells and leucocytes were found with the PVA meshes. Our microscopic results showed the macrophages could stimulate fibroblast in the fourth phase to improve the mature collagen deposition.

Results of tissue integration and mesh dislocation.

Ingrowth of the mesh into the surrounding area was excellent (tissue ingrowth of >75% of mesh) after 28 POD which was scored as 1. It was well‐integrated (up to 75% of the surface) on the 14th POD, which was scored as 2, whereas moderate integration (no tissue ingrowth, less than 50% of the surface) was in three cases in different termination days which was scored as 3.

All mesh and sutures were in place and no mesh dislocation was observed. In all cases the score was 1.

### 3.6 Histological evaluation

**FIGURE 6:**
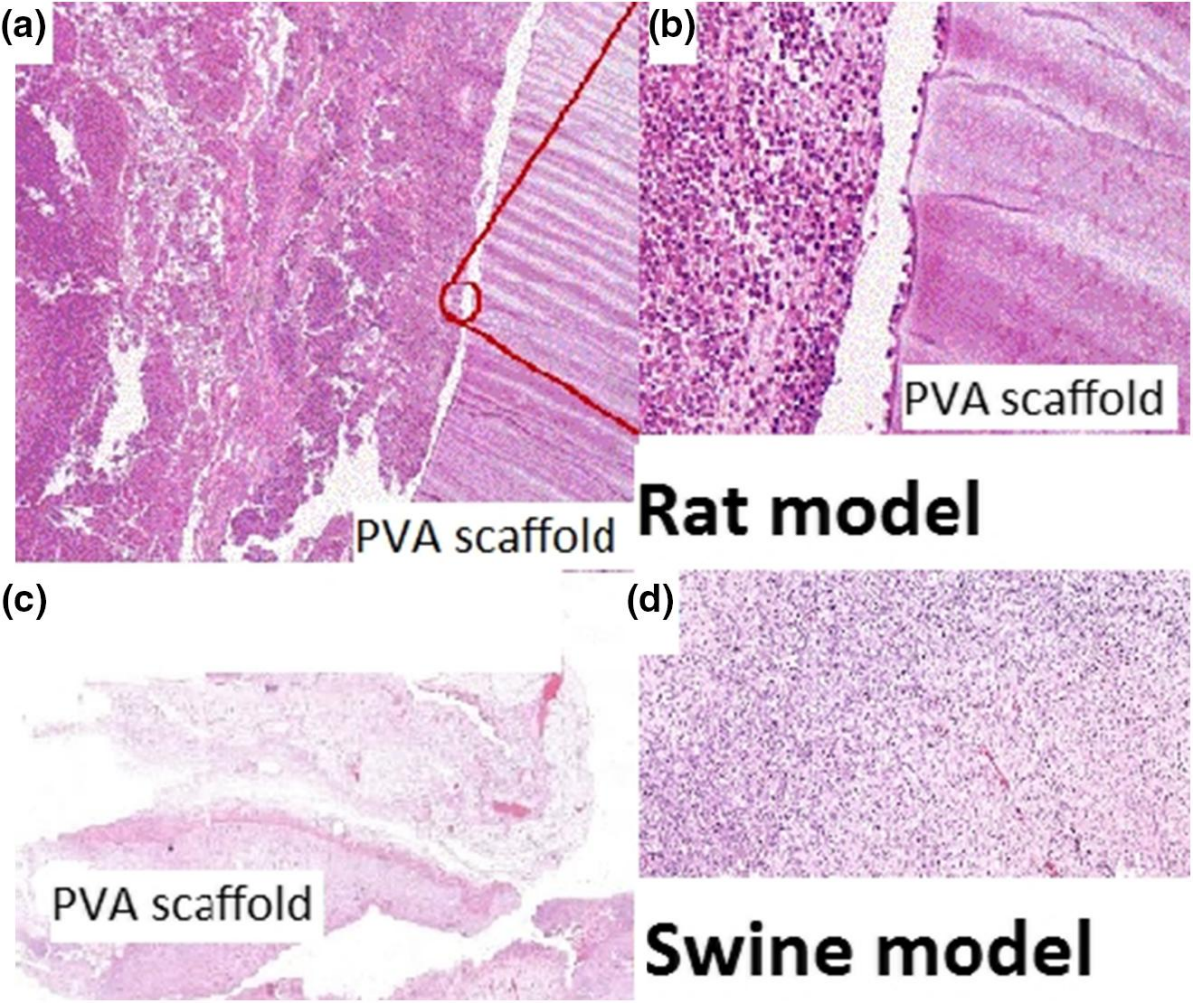
Representative images of H&E at the mesh‐tissue interface in rat model: PVA mesh on the 14th POD (a), cell migration into the PVA nanofibre (b), after 5 weeks PVA scaffold implantation in swine (c), some foreign giant cell on the mesh interface (d) red circles show foreign body giant cells

Upon histological evaluation in rats (Figure 6a,b), it was found that after one week we could observe inflammatory cells, neutrophils, and lymphocytes in surrounding the PVA scaffolds. After a period of 14 days the intensity of the inflammatory reaction decreases, some leucocytes are still present. On the 90th POD, granulation tissue has become mature fibrotic tissues (Figure 6b).

In pigs, after 5 weeks protocol period, implants were found surrounded by granulation tissue. Some inflammatory and giant cells are still present while fibroblasts started to produce collagen fibres. The results indicate similarity in all analysed samples. Comparing the semi‐quantitative histological parameters mentioned before, no significant differences were found between groups. Increased angiogenesis and less signs of inflammation was observed with the PVA mesh (Figure 6c,d), with increased oedema and adhesion formation in animals with the PP mesh.

## 4 DISCUSSION

Hernia mesh has a specialized function in the healing process after its implantation. Nowadays commonly used non‐degradable surgical meshes often cause serious complications, such as infection, adhesion formations, mesh migration, chronic pain, moreover intestinal perforation. To prevent these side effects, researchers work hard to develop new biocompatible and biodegradable synthetic materials [24, 25]. For instance, the ideal mesh still has not been developed. Biodegradable plastics and polymers were introduced in 1980s, there are two varieties on the market, synthetic and natural polymers. The biodegradation of these synthetic polymers takes place through the fragmentation of the polymer or degradation by microorganism, depends on their chemical structures [26, 27], and the processing characteristics [28, 29]. Most of the polymers are degrades through an oxidation process, contain an oxidizable functional group [30]. Polyvinyl alcohol is widely used because of its solubility in water. It can be easily degrading by microorganisms as well as enzymes [31]. Thereby medicine is one of the main sectors where biodegradable polymers have been introduced. Biodegradable polymers applications include not only pharmacological devices, as matrices for enzyme immobilization and controlled‐release devices [32] but also therapeutic devices for tissue engineering. Biodegradable biomaterials have been recently reviewed [33, 34, 35]. The application of synthetic biodegradable polymers dates back in the 1950s. These materials should have three important properties: biocompatibility, bioabsorbability and mechanical resistance. Biodegradable polyesters are widely employed as porous structure in tissue engineering because they typically have good strength and an adjustable degradation speed [36, 37]. These materials have received more attention in the last decades. Recently, numerous nanofibres have been investigated and widely used in biomedical applications, because of their unique properties, for example, porous structure which can support cells and mimics the extracellular matrix [38, 39]. To improve the properties of biodegradable polymers, a lot of methods have been developed. The future outlook for development in the field of polymer materials is promising. To improve the properties of biodegradable polymers, a lot of methods have been developed. These methods improve both the biodegradation rate and the mechanical properties of the final products. Physical blending is another route to prepare biodegradable materials with different morphologies and physical characteristics. Nano‐biocomposites are still under investigation. The use of biodegradable scaffolds has been increasing in recent years. A variety of devices (stents, artificial organs, biosensors, scaffolds for tissue engineering, etc.) have been developed for implantation into patients, unfortunately, most of them do not perform as well as expected. Laparoscopic surgery has allowed the introduction of new techniques for the repair of abdominal wall defects, minimising adhesion formation [40]. Surface effects must be considered a significant set of functional nanoproperties from the cell viability to laparoscopically implanted scaffold structure, degradation or cytotoxicological profile. Our research focuses on detailing the electrospun PVA hernia mesh structure, toxicity and degradation properties through cell viability, proliferation, and animal studies. Every mesh has different properties, for example, degradation time or shrinkage [41]. This study examined the biocompatibility in cell lines of our PVA nanofibre meshes which were produced by electrospun method. After cross‐linking, we have studied the changes in the morphologies of the cells on the nanofibres.

The sensitivity of this study based on observation of the cell morphology. In that case, the most important criteria for polymer materials are their cytotoxicological profile. To determine toxicity of the PVA solution in different concentration ratio and to study the adherence and morphology behaviour of the cells, hydrogel and scaffold formats were used. Cell viability and proliferation test were taken on HDFa cell line. For cell investigation, cells were cultured on 24 wells plates. On PVA nanofibre scaffolds, which were inoculated with HDFa cell culture, analyses were done to view the morphology of the attached cells on the scaffolds. The cells were painted for the visualization with Vybrant Dio. The in vitro experiments showed that the cells could not attach to the surface, but they could be found in the edge of the meshes. There are numerous investigations on the mechanical properties, but there is no description like our research which is focussing on the performance as a hernia mesh. The evaluation of biological effects of intraperitoneal positioned PVA fibres should also be considered as most significant that requires careful characterization through adhesion formation in the living system. In our animal studies, PVA scaffolds were used to reconstruct the abdominal wall in small and large animal models. While the small animal model (with Wistar rats) experiments were carried out to see tissue response and long‐term stability, the large animal model (with swine) was used to determine the anti‐adhesive property of the PVA membrane and to investigate the biocompatibility by measuring the signs of adhesion formation and inflammatory response through the connective tissue determination.

Swine is being used as a surgical model in medical research over the last 20 years [42, 43]. This animal model has a lot of similarity in anatomic and physiologic characteristics with humans. Pig skin is structurally like human epidermal thickness and dermal–epidermal thickness ratios. They could be a standard model of wound healing in regenerative medicine because of their blood supply in the cutaneous [44].

It was observed that the PVA scaffolds have essential anti‐adhesive properties. In the long‐term animal model, there was likely a direct interaction between the PVA scaffold, as the extracellular matrix, and the tissue on both sides of the lesion. These structures create a scaffold that connects to the two faces of the lesion, allowing movement of cells into the scaffold. The PVA mesh in our experiments created a permissive environment for growth while discouraging or preventing the scar formation which normally occurs at the early stage. The scaffolds had normal physiology and could integrate to the surrounding tissues, and we still could not find adhesion on the surface of the samples. These data support recent pilot study with large animal model, where we studied integration and biomechanical effects of abdominal wall reconstructive surgery with two different meshes. We challenged new mesothelial layer with minimal foreign body reaction around the PVA scaffolds. The mechanism of this phenomenon plays an important role in the incorporation process. Histological evaluation showed that vascularization and angiogenesis started as we found fibroblast produced collagen fibres in the surrounding area of the samples, demonstrating cell migration onto the edge of the nanofibres. In all cases, meshes were well integrated after the short‐term and long‐term experiments in the small animals, and in the 5‐weeks period with swine also. Biomaterials should provide mechanical strength until sufficient mature neo‐tissue and vascularization are formed.

Systematic reviews show significant correlation between incorporation and adhesion formation [45]. In that case, number of the animals should be raised for further information. Suturing also leaves scaffolds with defects, which become the samples main weak points [46]. Our research group demonstrated that suture line could be a huge impact in hernia surgery. The current studies analysed PVA mesh in different animal models in terms of adhesion formation and incorporation after a short and long‐term period. This paper mainly presents the preparation of electrospun poly(vinyl alcohol) fibre membranes and their in vitro and in vivo behaviours. This PVA meshes show good biocompatibility and biodegradability and the useability of their in vivo anti‐adhesion properties is presented. The animal experiments demonstrated that PVA meshes can integrate into the surrounding area with minimal inflammatory reaction.

The novelty in this paper is the microstructure of the PVA mesh implanted to the swine would promote good mechanical properties as a hernia mesh and could be an anti‐adhesive barrier, which is an important role in hernia surgery. For these parameters PVA mesh showed good results compared with PP mesh.

## 5 CONCLUSION

In summary we conclude that the PVA scaffolds were not toxic in the living systems. Animal studies indicate great potential for tissue engineering, especially for hernia repair.

## FUNDING

This study was supported by Hungarian Scientific Research Fund (Grant OTKA K105523, K115259).

## CONFLICT OF INTEREST

The authors declare that the research was conducted in the absence of any commercial or financial relationships that could be construed as a potential conflict of interest.

## PERMISSION STATEMENT

None.

6

### DATA AVAILABILITY STATEMENT

The data that support the findings of this study are available on request from the corresponding author. The data are not publicly available due to privacy or ethical restrictions.
